# Psycho‐Spiritual Challenges Faced by Palliative Health Care Providers in Bangladesh: A Multicenter‐Based Descriptive Cross‐Sectional Study

**DOI:** 10.1002/hsr2.70574

**Published:** 2025-03-18

**Authors:** Mastura Kashmeeri, A. N. M. Shamsul Islam, Palash Chandra Banik

**Affiliations:** ^1^ Department of Public Health & Hospital Administration NIPSOM Dhaka Bangladesh; ^2^ Department of Noncommunicable Diseases Bangladesh University of Health Sciences (BUHS) Dhaka Bangladesh; ^3^ Center for Higher Studies and Research Bangladesh University of Professionals Dhaka Bangladesh

**Keywords:** challenges, healthcare providers (HP), palliative care, psychological stress, psycho‐spiritual challenges, spiritual level

## Abstract

**Background:**

Palliative care providers play a crucial role in supporting patients with life‐limiting illnesses by emphasizing the alleviation of suffering and enhancing quality of life. The intrinsic nature of palliative care, which often involves confronting death and dying, places considerable emotional and spiritual demands on caregivers. Psycho‐spirituality, an integration of psychological well‐being and spiritual dimensions, is vital for these providers to maintain their mental health and provide holistic care to their patients. Despite its importance, empirical studies focusing on the psycho‐spiritual experiences of palliative care providers are limited.

**Method:**

This multicenter‐based, descriptive cross‐sectional study aims to explore the psycho‐spiritual challenges faced by palliative care providers. Data was collected from 160 licensed healthcare providers, through face‐to‐face semi‐structured interviews conducted from August to September 2022.

**Result:**

The results indicate that younger healthcare providers (aged 20–39) report higher psychological stress compared to their older counterparts (*p* = 0.029). Males exhibited slightly higher stress levels than females (*p* = 0.036), and divorced individuals reported the highest stress levels (*p* = 0.01). Educational qualification (*p* = 0.017), and income levels (*p* = 0.001) showed significant correlations with spiritual status with higher educational attainment and income associated with better spiritual well‐being. Doctors experienced higher psychological stress (*p* = 0.004) but also reported higher spiritual status compared to nurses and other healthcare workers.

**Conclusion:**

The findings depict significant associations between sociodemographic factors and the psychospiritual well‐being of healthcare providers. Understanding these associations is crucial for developing targeted interventions to support the well‐being of palliative care providers, ultimately leading to better patient care outcomes. Future research should focus on expanding the scope.

**Abbreviations**
PC: palliative care
PCA: palliative care assistant
WS: ward staff

## 1 Background

Palliative care providers play a critical role in supporting patients with life‐limiting illnesses, emphasizing the alleviation of suffering and the enhancement of quality of life. The nature of palliative care, which often involves confronting death and dying, places considerable emotional and spiritual demands on caregivers. Psycho‐spirituality, a concept that integrates psychological well‐being with spiritual dimensions, is crucial for these providers to maintain their own mental health and provide holistic care to their patients [1, 2]. Research has highlighted that palliative care professionals frequently encounter significant psychological stressors, including burnout, compassion fatigue, and moral distress [3]. These stressors are compounded by the need to engage deeply with patients' and families' spiritual concerns, which can profoundly affect providers' own spiritual and existential beliefs [4]. Consequently, there is a growing recognition of the importance of addressing the psycho‐spiritual needs of palliative care providers to ensure their well‐being and sustain their ability to offer compassionate care. Despite the acknowledged importance of psycho‐spirituality in palliative care, empirical studies specifically focusing on the psycho‐spiritual experiences of palliative care providers remain limited. Most existing research has been conducted in single‐center settings or within specific cultural contexts, limiting the generalizability of findings [5]. In the *Introducing Palliative Care* (5th edition) by Robert Twycross, spirituality is defined as: “Spirituality is that which gives meaning and purpose to life and relates to the way people experience their connectedness to the moment, to self, to others, to nature, and to the significant or sacred.” This definition emphasizes spirituality as a broad, individual, and deeply personal concept, encompassing a sense of purpose, interconnectedness, and the search for meaning, which are particularly important in the context of palliative care [6, 7, 8].

This multicenter‐based, descriptive cross‐sectional study aims to fill this gap by exploring the psycho‐spiritual challenges faced by palliative care providers across diverse settings. This study will investigate how palliative care providers across multiple centers perceive and experience psycho‐spiritual challenges. It will examine the prevalence of psychological and spiritual distress, the coping mechanisms employed, and the support systems in place to mitigate these challenges. By providing a comprehensive understanding of these issues, the study seeks to inform interventions that can enhance the well‐being of palliative care providers, ultimately leading to better patient care outcomes.

## 2 Methods

### 2.1 Study Design and Study Settings

In this research, a descriptive cross‐sectional study census method was used. Data was gathered from BSMMU, Dhaka College & Hospital, Delta Medical College & Hospital, National Institute of Cancer Research Hospital, and community‐based palliative care projects of BSMMU in collaboration with WHPCA in Korail and Narayanganj City Corporation.

### 2.2 Study Population and Sample Size

It involved 160 participants, of which 52 were Doctors, 64 were nurses, PCA (palliative care assistants) & ward staff number was 34, rest 8 were administrative staff, selected using the census method from an estimated staff population of 178, 5 of them were on leave and rest 13 healthcare providers refused to participate in the data collection process. Data collection occurred from August to September 2022. The study targeted licensed healthcare providers of both sexes, including doctors, nurses, palliative care assistants, ward staff, and PCAs.

### 2.3 Inclusion and Exclusion Criteria

The participants who were providing palliative care for at least 1 year in these four healthcare facilities in Dhaka City, Bangladesh were included in the study. Providers who were physically or mentally unfit, were in leave or were unwilling to participate remain excluded from the study.

### 2.4 Data Collection Process and Analysis

Data collection involved internally validated, self‐administered semi‐structured questionnaires & face to face interviews conducted based on predetermined variables. The questionnaire was originally developed in English and then translated into Bangla using some tools such as the perceived stress scale (PSS) & The 29‐item Spiritual Intelligence Questionnaire, which were valid in English. Interviews, conducted privately, lasted about 30 min each, and participation was voluntary. Data processing involved categorizing, coding, summarizing, and entering data into SPSS software. Categorical and numerical variables, by using a 5‐point Likert scale, were treated separately: a “Never” or “completely disagree” response was scored as “0,” a “Almost never” or “disagree” as “1,” “Sometimes” or “Almost agree” as “2,” “Fairly often” or “Almost agree” as “3” and “Very often,” or “Completely agree” as “4.” There were 21 questions for measuring the PSS; the lowest score is 0, and the highest is 84, which was further divided into three categories, depending on their Mean ± SD, Mean − 1 SD = High Stress (upto 35.87), Mean + 1 SD = Low psychological stress (48.75 and above), the score in between them are considered as average psychological stress (35.88 to 48.74). Out of 29 items on the Spiritual Intelligence Questionnaire, only 11 questions were used; the maximum score was 44, and the lowest score was 0, depending on their Mean ± SD, which was further categorized into poor (Mean − 1 SD = upto 15.76), average (15.77–28.91) and High (28.92 and above). Descriptive statistics were used for qualitative and quantitative variables, while inferential statistics were applied to determine relationships among variables.

### 2.5 Ethical Considerations

Ethical approval was obtained from the Institutional Review Board (IRB) of NIPSOM (Memo no: NIPSOM/IRB/2017/09), and verbal and written permissions were granted by the hospital's director. Participants provided informed consent, and their confidentiality was strictly protected. The investigators ensured privacy and secure storage of data, limiting transfer and ensuring no sharing with non‐study personnel.

## 3 Results

A total of 160 responses were received, completed, and analyzed, resulting in a response rate of 89.9%. The socio‐demographic characteristics of the respondents reveal a diverse group primarily composed of individuals aged 20–39 years (66.3%), with a mean age of 33.59 ± 8.050. The majority of respondents are female (68.1%), married (84.4%), and predominantly follow Islam (84.4%). Educationally, more than half (52.5%) have completed graduation, while 24.4% hold postgraduation degrees. In terms of occupation, nurses represent the largest group (40%), followed by doctors (32.5%). Regarding designation, the most common role is that of Senior Staff Nurse (37.5%), with a significant portion having 4–6 years of experience in their designated post (67.5%). The analysis of psychological and spiritual states indicates that most healthcare providers are in an average psychological state (60.6%) and spiritual status (63.1%) (Table 1).

**TABLE 1:** Distribution according to socio‐demographic traits.

Sociodemographic traits of respondents		
Age range	Frequency	Percent
20–39 years	106	66.3
40–59 years	54	33.8
Mean age	33.59 ± 8.050	
Sex		
Male	51	31.9
Female	109	68.1
Religion		
Islam	135	84.4
Hinduism	22	13.8
Christianity	3	1.9
Marital status		
Married	135	84.4
Unmarried	19	11.9
Divorced	6	3.8
Specialty of Physician		
Anesthesiology	6	3.8
Palliative Medicine	19	11.9
Internal Medicine	6	3.8
Radiation oncology	6	3.8
Public Health	4	2.5
No‐specialization	10	6.3
Not applicable (not physicians)	109	68.1
Working experience in Specialty		
1–3 years	31	19.4
3–6 years	11	6.9
> 6 years	12	7.5
Not applicable (not physicians)	109	66.3
Designation		
Senior doctors (Asst. prof/Assoc. prof/Professor)	5	3.1
Junior doctors (Medical officer/Resident doctor)	38	23.8
SSN	60	37.5
PCA/Ward staff + Junior Staff	34	21.2
Admin staff	23	14.4
Junior staff	13	8.1
Working experience in the designated post		
1–3 years	53	33.1
4–6 years	55	67.5
7–9 years	24	82.5
10–above	28	17.5
Overall psychological state		
Poor (high psychological stress)	51	31.9
Average (average psychological stress)	97	60.6
Good (poor psychological stress)	12	7.5
Overall spiritual level		
Poor spiritual level	23	14.4
Average spiritual level	101	63.1
High spiritual level	36	22.5
Income		
< 20,000	48	30
20,000–40,000	39	24.4
41,000–60,000	20	12.5
61,000–80,000	20	12.5
81,000–1 lakh (BDT)	33	20.6
Category of hospital working for		
Medical College	47	29.4
Specialized hospital	37	23.1
BSMMU (Hosp)	53	33.1
Community‐based treatment center	23	14.4

There are significant associations between socio‐demographic traits and these states. For instance, younger respondents (20–39 years) report higher psychological stress compared to those aged 40–59 years (*p* = 0.029). Similarly, males exhibit slightly higher psychological stress levels than females (*p* = 0.036). Marital status also affects psychological stress, with divorced individuals reporting the highest stress levels (*p* = 0.01). Educational qualification correlates with spiritual status, as those with higher secondary education report higher spiritual status (*p* = 0.017). Occupation and designation are significant, with doctors experiencing higher psychological stress (*p* = 0.004) and higher spiritual status (*p* = 0.001). Lastly, income levels show that higher‐income groups (> 81,000 BDT) report better spiritual status (*p* = 0.004), while expenditure patterns also influence psychological and spiritual states, albeit less significantly. The analysis reveals significant associations between various sociodemographic factors and both psychological stress and spirituality among health‐care providers. Age, sex, marital status, education, occupation, designation, and income show notable differences in levels of psychological stress and spirituality. Understanding these associations can inform targeted interventions to support the well‐being of health‐care providers (Table 2).

**TABLE 2:** Differentiation between sociodemographic and psycho‐spirituality of health care providers (*N* = 160).

Variable	Psychological stress			Spiritual level		
Age range	*N*	Mean	SD	*N*	Mean	SD
20–39	106	1.83	0.560	106	2.11	0.637
40–59 & above	54	1.61	0.96	54	2.02	0.532
** *p*‐value	0.029	0.023	0.027	0.015	0.350	0.322
Sex						
Male	51	1.84	0.543	51	2.14	0.633
Female	109	1.72	0.595	109	2.06	0.591
** *p*‐value	0.036	0.196	0.182	0.217	0.424	0.436
Marital status						
Married	135	45.15	6.833	135	2.06	0.608
Unmarried	19	47.11	4.581	19	2.05	0.524
Divorced	6	55.17	4.956	6	2.67	0.516
* *p*‐value	0.01	0.445	0.026	0.052	0.016	0.041
Education						
Below secondary	3	43.33	3.215	3	1.33	0.577
Secondary level	13	44.38	5.237	13	1.69	0.855
Higher Secondary	22	47.32	5.304	22	2.23	0.528
Graduation	84	45.23	6.813	84	2.13	0.510
Postgraduation	38	46.68	8.101	38	2.08	0.673
* *p*‐value	0.517	0.999	0.735	0.017	0.009	0.038
Occupation						
Doctor	52	48.48	7.155	52	2.37	0.525
Nurse	64	44.72	6.957	64	2.13	0.488
Ward staff & PCA	36	44.39	5.505	36	1.75	0.649
Admin & office staff	8	42.50	2.563	8	1.38	0.518
* *p*‐value	0.004	0.014	0.024	0.001	0.006	0.002
Designation						
MO/Resident doctor	38	49.89	6.451	38	2.42	0.500
Asst. prof/Assoc. prof/Professor	5	52.20	7.463	5	2.40	0.548
SSN	60	45.02	7.075	60	2.13	0.503
Ward staff & PCA	21	41.57	4.935	21	1.38	0.498
Admin & office staff	23	41.43	3.259	23	1.83	0.576
Cleaner	13	49	3.055	13	2.31	0.480
* *p*‐value	0.001	0.002	0.005	0.001	0.008	0.001
Working experience in palliative care						
1–3 years	53	46.79	6.452	53	2.19	0.557
4–6 years	55	46.36	6.334	55	2.09	0.617
7–9 years	24	42.54	8.097	24	2.00	0.417
10–above	28	45.36	6.679	28	1.93	0.766
* *p*‐value	0.067	0.53	0.97	0.272	0.926	0.974
Category of hospital working for						
Medical college	47	46.38	6.045	47	2.15	0.551
Specialized hospital	37	45.54	6.314	37	2.03	0.600
BSMMU (Hosp)	53	46.64	8.369	53	2.30	0.463
CBT	23	42.78	3.988	23	1.52	0.665
* *p*‐value	0.126	0.941	0.872	0.001	0.001	0.020
Specialty of physician						
Anesthesia	6	1.83	0.753	6	2.00	0.632
Palliative medicine	19	2.11	0.567	19	2.58	0.507
Internal medicine	6	1.83	0.753	6	2.33	0.516
Radiation oncology	6	1.83	0.753	6	2.17	0.408
Public health	4	2.00	0.816	4	2.00	0.001
Non‐specialization	10	1.80	0.422	10	2.50	0.527
Not applicable	109	1.67	0.545	109	1.94	0.591
*p*‐value*	0.106	0.950	1.000	0.001	0.310	0.949
Working experience in specialty						
1–3 years	31	48.65	6.504	31	2.23	0.497
3–6 years	11	47.55	7.660	11	2.55	0.522
> 6 years	12	48.83	8.233	12	2.50	0.522
Not applicable	109	44.38	6.297	106	1.94	0.599
*p*‐value*	0.004	0.964	0.010	0.001	0.036	005
Income						
< 20,000	48	1.81	0.445	48	2.06	0.598
20,000–40,000	39	1.59	0.549	39	1.79	0.615
41,000–60,000	20	1.75	0.550	20	2.15	0.587
61,000–80,000	20	1.80	0.768	20	2.25	0.550
81,000–1 lakh & rest	33	1.85	0.667	33	2.30	0.529
*p*‐value*	0.331	0.386	0.327	0.004	0.040	0.003
Expense						
< 10,000	77	1.75	0.517	77	2.03	0.648
10,000–30,000	55	1.84	0.631	55	2.16	0.570
31,000–50,000	15	1.67	0.488	15	2.13	0.352
51,000–70,000	10	1.40	0.699	10	1.90	0.738
71,000–rest	3	2.00	1.00	3	2.33	0.577
*p*‐value*	0.222	0.925	0.365	0.529	0.699	0.910

Abbreviation: CBT, community‐based treatment center

* ANOVA test.

** *t*‐test.

## 4 Discussion

The socio‐demographic profile of the respondents in this study provides significant insights into the workforce dynamics within the healthcare setting. The study's findings on the association between sociodemographic factors and psychospiritual well‐being among healthcare providers align with and extend existing literature on occupational stress and spirituality in healthcare settings. This discussion will compare the study's results with relevant research to provide a comprehensive understanding of these dynamics.

### 4.1 Age and Psychospiritual

The high percentage of young adults aged 20–39 (66.3%) is in line with global healthcare trends, where a significant portion of the workforce is made up of younger professionals due to the demanding nature of healthcare jobs and the continuous influx of new graduates [9]. This youthful demographic suggests potential for innovation and adaptability, but it may also indicate a need for structured mentorship and career development programs to effectively harness their potential [10]. The study revealed that younger healthcare providers (aged 20–39) experienced higher levels of psychological stress compared to their older counterparts (aged 40–59 and above), with significant differences (*p* = 0.029). This is consistent with previous research indicating that younger professionals often face greater stress due to less experience and higher workload pressures [11]. For instance, a study by Shanafelt et al. (2015) also reported higher burnout rates among younger physicians, attributing it to factors such as work‐life imbalance and career uncertainty [12].

### 4.2 Gender Differences

The gender distribution shows a significant female majority (68.1%), which is in line with the global healthcare trend where women outnumber men, especially in nursing and allied health professions [13, 14]. This demographic characteristic highlights the need for workplace policies that support work‐life balance and address specific challenges faced by female healthcare workers, such as maternity leave and career progression barriers [15]. The study found significant gender differences in psychological stress, with males reporting higher stress levels (*p* = 0.036). This aligns with a previous study by Purvanova and Muros (2010) which found that male healthcare providers often experience higher stress due to societal pressures and expectations to excel in their careers [15]. However, there were no significant differences in spirituality between genders in this study (*p* = 0.424), which contrasts with some literature suggesting that women often report higher spiritual well‐being, possibly due to different coping mechanisms and social support networks.

### 4.3 Marital Status and Psychospiritual Well‐Being

The study highlights that divorced healthcare providers report the highest levels of psychological stress and spirituality. This is supported by research indicating that marital status can significantly impact stress levels, with divorced individuals often experiencing higher stress due to personal and professional life disruptions (*p* = 0.01). A study by Batool A et al. (2014) supports this, showing that marital instability can exacerbate stress and influence spiritual practices as coping strategies [16].

### 4.4 Educational Attainment

Higher educational levels were associated with increased spirituality, but not significantly with psychological stress. Post‐graduates reported higher spirituality scores (*p* = 0.009), possibly reflecting broader access to spiritual and professional development resources. This correlates with research by Tuck et al. (2001), which found that higher education levels often correlate with greater engagement in spiritual practices due to increased awareness and value placed on holistic well‐being [17].

### 4.5 Occupational Roles

According to professional designation data, Specific Staff Nurses make up the largest group (37.5%), while senior doctors are underrepresented (3.1%). This disproportionate distribution towards mid‐level healthcare workers emphasizes the crucial role that nurses and junior doctors play in delivering patient care. It also highlights the need for continuous professional development and opportunities for career advancement to retain and motivate these essential personnel [18]. The significant variations in psychological stress and spirituality among different occupational roles, with doctors reporting the highest levels, align with existing literature on occupational stress. Doctors typically face higher demands and expectations, leading to greater stress and potentially a higher engagement in spiritual activities as a coping mechanism (*p* = 0.004 for stress and *p* = 0.001 for spirituality). Shanafelt et al. (2012) also reported that physicians often turn to spirituality to mitigate the effects of burnout [12].

### 4.6 Experience in Palliative Care

The working experience data, with the majority having 4–6 years of experience (67.5%) and a significant number with 7–9 years (82.5%), indicates a relatively experienced workforce. This level of experience is beneficial for maintaining high standards of patient care and operational efficiency. However, the presence of a substantial number of less experienced staff (1–3 years: 33.1%) calls for robust onboarding and training programs to ensure they quickly reach the competency levels required for effective service delivery [19]. No significant association was found between experience in palliative care and psychological stress or spirituality (*p* = 0.067 and 0.926, respectively). This finding contrasts with some studies suggesting that longer experience in palliative care can lead to better‐coping mechanisms and lower stress levels due to increased resilience and professional support systems. However, it aligns with research by Peters et al. (2013) indicating that the emotional demands of palliative care can consistently affect practitioners regardless of their experience level [20].

### 4.7 Income and Expenses

Income distribution reveals that a significant portion of the workforce earns less than 20,000 BDT (30%), with a notable fraction in the 20,000–40,000 BDT range (24.4%). This reflects the economic challenges faced by many healthcare workers, which can impact their overall job satisfaction and quality of life [21]. Improving compensation and benefits could be a strategic approach to enhance workforce stability and performance. Higher‐income levels were associated with higher spirituality scores, suggesting that financial stability may allow healthcare providers more resources and time to engage in spiritual practices (*p* = 0.003). This is supported by research indicating that financial security can enhance overall well‐being, including spiritual well‐being, by reducing stress and enabling access to spiritual resources. However, the lack of significant associations between expenses and psychospiritual factors (*p* = 0.222 and 0.529) suggests that financial outflow does not directly impact these aspects, which might indicate that personal financial management strategies vary widely among individuals [20, 21].

### 4.8 Psycho‐Spiritual Challenges Faced by Palliative Healthcare Providers in Bangladesh

#### 4.8.1 Psychological Stress and Occupational Roles

The study identifies significant occupational disparities in stress and spirituality levels. Doctors report the highest psychological stress (*p* = 0.004), potentially stemming from their dual responsibilities of patient care and decision‐making in life‐limiting conditions. This echoes the findings by Kearney et al. (2009), who emphasize that physicians in palliative care often experience “moral distress” due to the emotional weight of end‐of‐life decision‐making [3, 6, 7, 8, 22, 23].

Conversely, administrative staff and junior‐level workers report lower stress levels but also lower spiritual engagement (*p* = 0.001). This divergence might stem from differences in job roles, with front‐line staff having more opportunities to connect spiritually with patients [4]. Nurses, forming 40% of respondents, experience moderate stress but relatively higher spiritual engagement, supporting Wilfred's (2006) model, which posits that spiritual care is integral to nursing and mitigates burnout [23, 24].

### 4.9 Education, Spirituality, and Well‐Being

Education levels correlate significantly with spiritual well‐being. Respondents with higher secondary education report the highest spiritual levels (*p* = 0.017), consistent with Puchalski (2013), who argues that higher education fosters awareness of spiritual dimensions in patient care [1]. Notably, individuals with postgraduate qualifications display moderate stress, suggesting that advanced training might equip them with better coping skills or spiritual frameworks [25].

Income also significantly impacts spirituality. Higher‐income groups (>81,000 BDT) report better spiritual well‐being (*p* = 0.004), reflecting findings by Goodman and Schorling (2012), who argue that financial stability alleviates work‐related stress, allowing individuals to engage more deeply with spiritual care [26].

### 4.10 Workplace Environment and Psycho‐Spiritual Challenges

The type of healthcare facility impacts stress and spirituality levels. Community‐based treatment centers show the lowest spiritual engagement among workers (*p* = 0.001). This finding is alarming, given the pivotal role of spirituality in home‐based and community palliative care [8, 22]. In contrast, institutions like BSMMU report higher spiritual levels, possibly due to better infrastructure and training opportunities, as noted by Kashmeeri et al. (2024) [27].

Specialization in palliative medicine correlates with higher spirituality levels (*p* = 0.001). This reinforces the need for structured spiritual care training in medical education [1]. However, non‐specialized healthcare workers show relatively low spiritual engagement, indicating a gap in spiritual care integration at lower training levels [28].

### 4.11 Comparison With Global Literature

Globally, studies emphasize the intersection of psychological stress and spirituality in palliative care. For instance, Vallurupalli et al. (2012) found that spiritual engagement significantly enhances the quality of life for both patients and providers [29]. Similarly, Lizano et al. (2019) highlight that spiritual care reduces burnout and improves job satisfaction among healthcare workers [30].

However, the unique socio‐cultural context of Bangladesh presents additional challenges. Islamic traditions dominate the spiritual outlook of respondents (84.4% Muslim), influencing their engagement with spirituality in patient care. As highlighted by D'Andria Ursoleo et al. (2024), spirituality in predominantly Islamic settings often intertwines with community and familial support, creating both opportunities and challenges for healthcare providers [31].

The biopsychosocial‐spiritual model provides a comprehensive framework to address these challenges. This model emphasizes the integration of spirituality into holistic care, resonating with the findings that spiritual well‐being mitigates stress and enhances professional satisfaction [6, 7, 8, 22, 23].

### 4.12 Implications for Policy and Practice

Understanding the psycho‐spiritual challenges faced by palliative care providers in Bangladesh necessitates targeted interventions. Training programs should emphasize spiritual care as an essential skill for healthcare workers across all roles, as suggested by Twycross [6, 7, 8, 22, 23]. Regular spiritual assessments, akin to those outlined by Biswas et al. (2024), can help identify providers at risk of burnout and guide personalized interventions [32].

Moreover, workplace spirituality, as explored by Faro Albuquerque et al. (2014), should be fostered through supportive leadership and team‐based approaches. Spiritual leadership, in particular, has been shown to reduce burnout and enhance job satisfaction [11, 33]. Institutions must also address systemic stressors, such as low pay and inadequate training, which exacerbate psycho‐spiritual challenges.

## 5 Limitations

One notable limitation of this study is its reliance on self‐reported data collected through face‐to‐face semi‐structured interviews, which may introduce response bias. Participants might have provided socially desirable answers rather than reflecting their true experiences and feelings, potentially skewing the results. Additionally, the cross‐sectional nature of the study limits the ability to draw causal inferences about the relationship between sociodemographic factors and psychospiritual well‐being. The sample, while covering multiple centers, besides not including some autonomous centers providing care, is also geographically confined to Dhaka city, which may not fully represent the experiences of palliative care providers across Bangladesh. Consequently, the findings may lack generalizability to broader contexts within the country.

## 6 Recommendations

To address the limitations identified, future research should consider employing longitudinal study designs to better capture the dynamics of psychospiritual well‐being over time and establish causal relationships. Expanding the geographic scope of the study to include palliative care providers from autonomous sectors in Bangladesh can enhance the generalizability of the findings. Additionally, incorporating objective measures of psychological and spiritual well‐being, alongside self‐reported data, could mitigate response bias and provide a more comprehensive understanding of the challenges faced by healthcare providers. Implementing targeted interventions based on these expanded studies, such as structured mentorship programs for younger professionals and financial support initiatives, could significantly improve the overall well‐being and retention of palliative care providers.

## 7 Conclusion

The study underscores the intricate relationship between sociodemographic factors and the psychospiritual well‐being of healthcare providers. Age, gender, marital status, education, and occupational roles significantly influence psychological stress and spirituality, highlighting the need for tailored support systems in healthcare settings. These findings align with and extend existing research, providing a comprehensive view of the factors affecting healthcare providers' well‐being. Future studies could further explore these associations and develop targeted interventions to support this vital workforce.

## Author Contributions

**Mastura Kashmeeri:** conceptualization, methodology, data curation, software, investigation, validation, formal analysis, writing – original draft, writing – review and editing, resources, visualization, funding acquisition. **A. N. M. Shamsul Islam:** supervision, project administration, writing – review and editing. **Palash Chandra Banik:** writing – review and editing, formal analysis, supervision, validation.

## Conflicts of Interest

The authors declare no conflicts of interest.

## Transparency Statement

The lead author Mastura Kashmeeri affirms that this manuscript is an honest, accurate, and transparent account of the study being reported; that no important aspects of the study have been omitted; and that any discrepancies from the study as planned (and, if relevant, registered) have been explained.

## Supporting information

Supporting information.

## Data Availability

The data that support the findings of this study are openly available in DOI:10.17632/stxdn5cjhh.1 at https://data.mendeley.com/preview/stxdn5cjhh?a=88fbd17e-31ee-4817-b576-fbf1be3c2569, reference number Manuscript ID HSR‐2024‐06‐1738. All data relevant to the study are accessible in Mendeley data DOI:10.17632/stxdn5cjhh.1; https://data.mendeley.com/preview/stxdn5cjhh?a=88fbd17e-31ee-4817-b576-fbf1be3c2569.
